# Determining delayed admission to the intensive care unit for mechanically ventilated patients in the emergency department

**DOI:** 10.1186/s13054-014-0485-1

**Published:** 2014-08-23

**Authors:** Shih-Chiang Hung, Chia-Te Kung, Chih-Wei Hung, Ber-Ming Liu, Jien-Wei Liu, Ghee Chew, Hung-Yi Chuang, Wen-Huei Lee, Tzu-Chi Lee

**Affiliations:** Department of Emergency Medicine, Kaohsiung Chang Gung Memorial Hospital and Chang Gung University College of Medicine, Kaohsiung, Taiwan, No.123, Dapi Road, Niaosong District, Kaohsiung City, 833 Taiwan; Department of Internal Medicine, Kaohsiung Chang Gung Memorial Hospital and Chang Gung University College of Medicine, Kaohsiung, Taiwan, No.123, Dapi Road, Niaosong District, Kaohsiung City, 833 Taiwan; Department of Public Health, Kaohsiung Medical University, Kaohsiung, Taiwan, No. 100, Shihchuan 1st Road, Sanmin District, Kaohsiung City, 807 Taiwan; Department of Community Medicine, Kaohsiung Medical University Chung-Ho Memorial Hospital, No. 100, Tzyou 1st Road, Sanmin District, Kaohsiung City, 807 Taiwan

## Abstract

**Introduction:**

The adverse effects of delayed admission to the intensive care unit (ICU) have been recognized in previous studies. However, the definitions of delayed admission vary across studies. This study proposed a model to define ‘delayed admission’, and explored the effect of ICU waiting time on patients’ outcome.

**Methods:**

This retrospective cohort study included nontraumatic adult patients on mechanical ventilation in the emergency department (ED), from July 2009 to June 2010. The primary outcomes measures were 21-ventilator-day mortality and prolonged hospital stays (over 30 days). Models of Cox regression and logistic regression were used for multivariate analysis. The non-delayed ICU waiting was defined as a period in which the time effect on mortality was not statistically significant in a Cox regression model. To identify a suitable cutoff point between ‘delayed’ and ‘non-delayed’ subsets from the overall data were made based on ICU waiting time and the hazard ratio of ICU waiting hour in each subset was iteratively calculated. The cutoff time was then used to evaluate the impact of delayed ICU admission on mortality and prolonged length of hospital stay.

**Results:**

The final analysis included 1,242 patients. The time effect on mortality emerged after 4 hours, thus we deduced ICU waiting time in the ED of >4 hours as delayed. By logistic regression analysis, delayed ICU admission affected the outcomes of 21-ventilator-day mortality and prolonged hospital stay, with an odds ratio of 1.41 (95% confidence interval, 1.05 to 1.89) and 1.56 (95% confidence interval, 1.07 to 2.27) respectively.

**Conclusions:**

For patients on mechanical ventilation in the ED, delayed ICU admission is associated with higher probability of mortality and additional resource expenditure. A benchmark waiting time of no more than 4 hours for ICU admission is recommended.

## Introduction

Emergency department (ED) overcrowding has been a global issue, and is also getting more frequent in Taiwan [1,2], where there has been National Health Insurance (NHI) since 1995. Even critically ill patients are not spared, and often have to board in the ED while they wait for admission to intensive care units (ICUs) [3]. Regarding the issue of overcrowded EDs, critically ill patients expend a relatively high capacity of ED, leading to a reduced ED capacity for handling new arrivals. If the critically ill patients have to stay in the ED because of insufficient ICU beds, ED crowdedness would worsen [4,5]. In such a situation, the patients and his or her family are left suffering and dissatisfied [6]. The risks of medical errors are high, and patients’ safety may potentially be jeopardized [5,7].

Outcomes for critically ill patients, such as patients of acute respiratory failure with ventilator support, are influenced by whether or not optimal intensive care is delivered in a timely manner, which in turn is determined by how long the patient waits in the ED for ICU admission. The adverse effects of delayed ICU admission have been recognized in previous studies [8–11]. However, these studies treated ICU waiting time as a dichotomous variable, the definition of ‘delayed admission’ differed among them. In addition, these studies mentioned less about how to identify ‘delayed admission’, and the enrolled populations were mainly focused on those who had been admitted to the ICU. Although a few studies used length of time spent waiting in the ED prior to ICU admission as a variable to explore associated adverse outcomes [12], it has not been known how long waiting is delayed. Because the predicaments of ED overcrowding and subsequent ED boarding of critically ill patient are getting more prevalent, we would need a new model, which also involves the adverse events occurring in the ED before ICU admission, to demarcate the phases of delayed- and non-delayed ICU admission, and to be rendered as a benchmark for quality monitoring.

The object of this study was to attempt to find the optimal timing to demarcate the delayed and non-delayed, and explore the effect of delayed ICU admission on patients’ mortality and subsequent extra use of health-care resources if the patients survived the first 21 ventilator days.

## Materials and methods

The study was conducted under the approval of the institutional review board of Kaohsiung Chang Gung Memorial Hospital. The retrospective data was collected from past chart records, and the institutional review board approved waiving the need for informed consent.

### Settings and study design

This was a retrospective observational cohort study, conducted at Kaohsiung Chang Gung Memorial Hospital, a medical center in southern Taiwan with a capacity of 2,715 beds (with 206 ICU beds). The ICUs are categorized under surgery, medicine, and pediatrics. The ICU practice environment was a close system serviced by a fixed physician staff. Critically ill patients were admitted under the principle of ‘first come, first served’. The ED adopted a five-level triage system (that is, resuscitation, emergency, urgent, less urgent, and not urgent). At the ED, if patients suffered from respiratory failure and received mechanical ventilation support, they were transferred to the ICU as soon as possible. Patients on mechanical ventilation stayed at the ED only when there were not enough ICU beds. The ICU booking was then done after connecting the patient to a ventilator.

The ED had a designated area to provide care for the boarding patients who required continuous monitoring. The stretchers were equipped with portable monitors for blood pressure, respiratory rate, cardiac rhythm, and oxygenation. The patient’s vital signs were taken every 4 hours routinely and more frequently if needed. If there were insufficient ICU beds, the mechanically ventilated patients were transferred to this area to wait for ICU admission. While waiting, the patients remained under the care of the ED physicians. Only the ED attending physicians, also specialists in intensive care medicine and certified by the United Credentials Committee of Critical Care Medicine, were in charge of the care of these patients. They teamed up with senior resident doctors, respiratory therapists, social workers and nurses. The overall nurse-patient ratio was about three to six. However, a nurse was not allowed to care for more than three patients on mechanical ventilation.

### Inclusion and exclusion criteria

The study population was focused on the non-trauma adult patients who were on ventilator support at the ED. Patients of pediatric age, organ transplantation donors, or those with trauma-related etiologies, chronic ventilator dependence, out-of-hospital cardiac arrest (OHCA), or unexpected in-hospital cardiac arrest (IHCA), who failed to have sustained return of spontaneous circulation (ROSC) over 2 hours after resuscitation (format as Health Administrator requiring) were all excluded [13]. Patients on ventilators who were transferred in were also excluded due to unknown ventilator time.

### Data collection and definitions

All patients’ data were collected via review of chart records. The demographics (age and sex), vital signs (that is, systolic and diastolic blood pressure, pulse rate, respiratory rate, and Glasgow coma scale), triage results, chief complaints, laboratory findings of blood samples (that is, complete blood cell count, prothrombin time, arterial blood gas results, and levels of blood urea nitrogen, creatinine, sodium, potassium, glucose, albumin, and bilirubin) and baseline comorbidities (for example, malignancy, acquired immune deficiency syndrome, leukemia, multiple myeloma, lymphoma, immunocompromised status, heart failure, chronic lung disease, chronic kidney disease, liver cirrhosis, atrial fibrillation, diabetes mellitus, and hypertension), hospital discharge condition, length of ventilator use, and length of ICU and hospital stay were collected.

The scores of acute physiology and chronic health evaluation (APACHE II) were calculated to evaluate disease severity on the first ventilator day at the ED [14]. The principal etiologies of respiratory failure were classified with APACHE II diagnostic categories. Any unplanned ED revisit within 72 hours or readmissions within 14 days were also verified as these were audited indexes of quality of ED practice.

### Time spent waiting for ICU admission

The ICU booking was made after connecting the patient to a ventilator, so the time spent waiting for ICU admission was measured as the number of hours from the connection to a ventilator at the ED until admission to the ICU or until the ICU booking was cancelled. The latter category included patients who expired in the ED, those who were transferred to another hospital, those who left the ED against medical advice, or those who were successfully liberated from the ventilator in the ED.

### Outcome measures

The primary outcome measure was 21-ventilator-day mortality, while the secondary outcome measure was prolonged length of hospital stay (>30 days) [15]. The first 21 ventilator days were also the first phase of the NHI Integrated Delivery System for Respiratory Care. This study investigated if ICU waiting time had any effects on patients’ mortality and determined the dividing line of ICU waiting time between delayed and non-delayed. This dividing line was then used to re-check the impact of delayed ICU admission on mortality and resource utilization. Patients who survived the first 21 ventilator days were reviewed to determine if they utilized more health-care resources as a result of their prolonged hospitalization.

### Statistical analysis

Continuous variables of patients’ baseline characteristics were reported as mean ± standard deviation, while between-group (mortality vs. survival) comparisons were made using Student’s *t* test. Categorical variables were reported as numbers and as percentages, whereas between-group comparisons were made using the chi-square test and Fisher’s exact test. The time length variables of ED arrival to ventilator use, ventilator use to ED departure, and entire ED stay were reported as median and as interquartile (IQR) range, and between-group comparisons were made by Wilcoxon’s rank sum test.

The primary outcomes were analyzed by univariate and multivariate Cox regression model and the logistics regression model. The observed case events in the Cox analysis were the fatalities in the first 21 ventilator days. Those without observed case results were censored. Proportional assumption of Cox model was assessed by Kolmogorov-type supremum test.

The definition of the dividing line between ‘delayed’ and ‘non-delayed’ was proposed as the time when the effect of ICU waiting on mortality started to emerge. To determine this demarcation of time (non-delayed going to delayed), subsets from the overall data were made based on the different length of ICU waiting time, such as ‘<2 hours’, ‘<3 hours’, ‘<4 hours’ and so on. Iterative calculations with the Cox regression model were made to estimate the hazard ratio (HR) of ICU waiting time (by hours) on mortality in each subset analysis and to find when the time effect started to emerge.

Statistical significance was set at a two-tailed *P* <0.05. All variable analyses were performed by using the SAS 9.2 (SAS Institute Inc.*,* Cary*,* NC, USA)*.*

## Results

**Figure 1:**
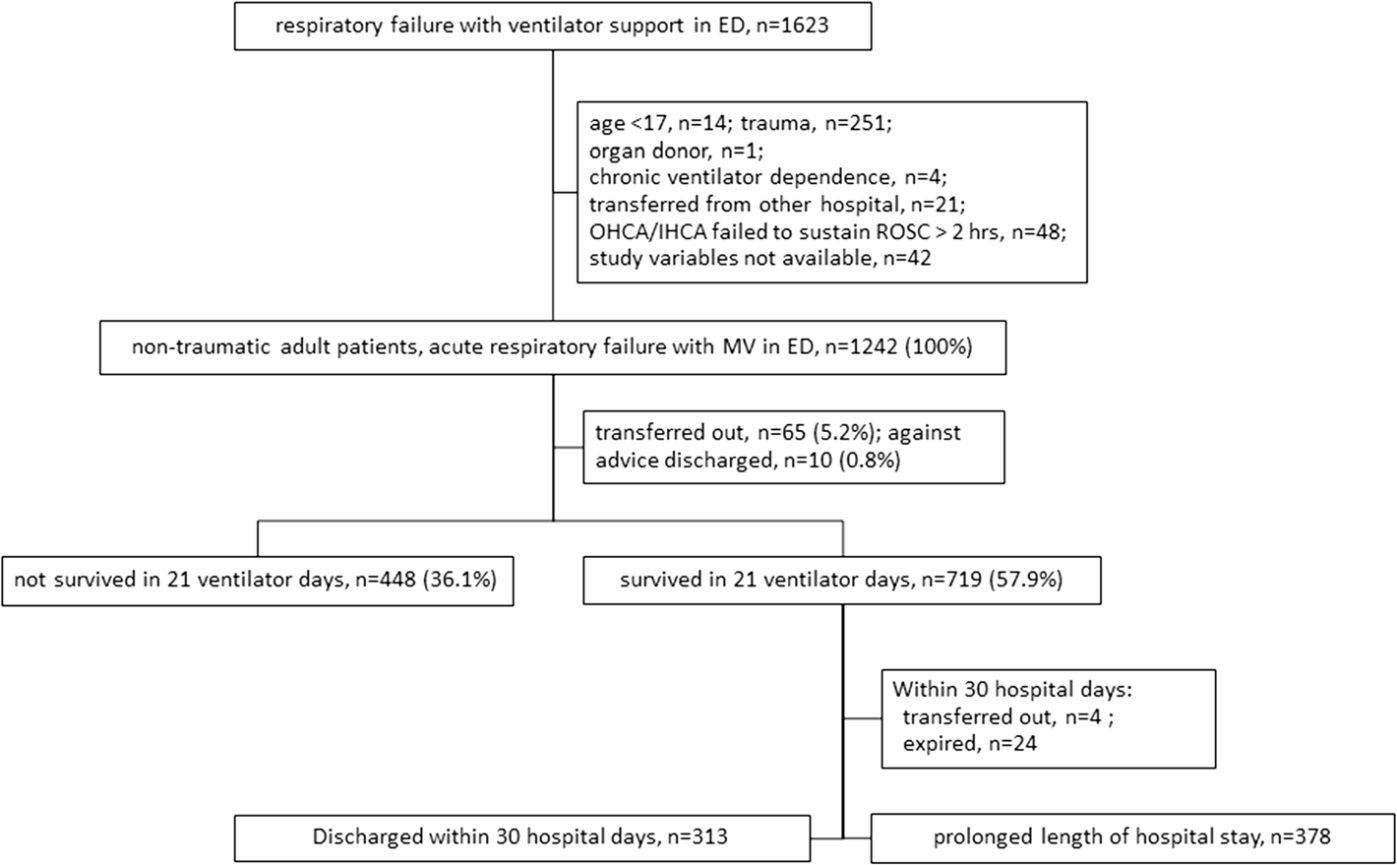
**Flow chart of enrolled patients.**

The ED of the study hospital received 132,221 patients during the study period, including 70,736 adult non-trauma patients. Among them, 1,623 patients suffered from acute respiratory failure due to various etiologies and received ventilator support at the ED. However, 14 patients aged <17 years, 251 trauma patients, 4 chronic respiratory failure patients who utilized a home ventilator, 1 organ transplantation donor, 21 who transferred from other hospitals, 48 OHCA/IHCA patients who failed to sustain ROSC over 2 hours, and 42 with incomplete APACHE II score were excluded (Figure 1). Thus, 1,242 non-trauma ventilated adult patients entered the study analysis.

By the patients’ baseline characteristics (Table 1), the mean age was 67.0 ± 15.4 years and 60.1% were male (n = 747). The overall mean APACHE II score was 22.3 ± 8.2. Analysis of triage records revealed that 656 patients (52.8%) were at level 1, 398 (32.1%) at level 2, and 188 (15.1%) at levels 3 to 5. Twenty-six patients (2.1%) had unplanned ED re-visits within 72 hours and 13 (1.1%) were re-admitted within 14 days of the previous discharge. The median time from the patient’s arrival at the ER to connection to a ventilator was 2.1 hours (IQR 0.4 to 11.6). The median time between connection to a ventilator and the patient’s leaving the ED was 10.2 hours (3.9 to 28.1). The median length of stay in the ED was 12.7 hours (6.8 to 49.9). The most frequent diagnostic category was respiratory infection (n = 343; 26.6%). The 14 most prevalent diagnostic categories accounted for 95.6% of all cases.

**Table 1:** **Baseline characteristics of patients**

		**Total (n = 1242) [** 1 **]**	**Transferred out or AAD (n = 75) [** 2 **]**	**Survived (n = 719) [** 3 **]**	**Not survived (n = 448) [** 4 **]**	***P*** **value [** 3 **] vs. [** 4 **]**	***P*** **value [** 1 **] vs. [** 2 **]**
**Age**		67.0 ± 15.4	66.1 ± 16.6	67.8 ± 15.5	66.0 ± 14.9	0.06	0.58
**Sex, male (%)**		747 (60.1)	48 (64)	412 (57.3)	287 (64.1)	0.02	0.48
**APACHE II score**		22.3 ± 8.2	22.8 ± 8.1	21.0 ± 7.6	24.1 ± 8.7	<0.01	0.56
**Triage (%)**	Level 1	656 (52.8)	47 (62.7)	370 (51.5)	239 (53.4)	0.33	0.16
	Level 2	398 (32.1)	21 (28.0)	243 (33.8)	134 (29.9)		
	Level 3,4,5	188 (15.1)	7 (9.3)	106 (14.7)	75 (16.7)		
**Unplanned ED Re-visit** ^***a***^ ***(%)***		26 (2.1)	0 (0)	14 (2.0)	12 (2.7)	0.41	0.19
**Re-admission** ^***b***^ ***(%)***		13 (1.1)	1 (1.3)	8 (1.1)	4 (0.9)	0.71	0.80
**ED arrival to ventilator, hours (Q1-Q3)**		2.1 (0.4-11.6)	1.8 (0.4-6.2)	2.0 (0.5-11.4)	2.6 (0.4-15.4)	0.44^***c***^	0.20^***c***^
**Ventilator to leaving ED, hours (Q1-Q3)**		10.2 (3.9-28.1)	12.9 (2.7-37.4)	10.8 (3.4-28.9)	9.7 (4.0-21.7)	0.02^***c***^	0.86^***c***^
**ED stay, hours (Q1-Q3)**		21.7 (6.8-49.9)	19.6 (5.7-53.0)	22.1 (6.5-49.7)	21.8 (7.4-49.7 )	0.07^***c***^	0.76^***c***^
**Principal diagnostic categories (%)**	Respiratory infection	343 (26.6)	21 (28)	220 (30.6)	102 (22.8)	<0.01	0.35
	Sepsis	274 (22.1)	12 (16)	124 (17.3)	138 (30.8)		
	ICH/SAH/SDH	113 (9.1)	8 (10.7)	61 (8.5)	44 (9.8)		
	CAD	73 (5.9)	3 (4.0)	53 (7.4)	17 (3.8)		
	CHF	58 (4.7)	0 (0)	50 (7.0)	8 (1.8)		
	Postcardiac arrest	56 (5.1)	2 (2.7)	15 (2.1)	39 (8.7)		
	COPD	47 (3.8)	4 (5.3)	34 (4.7)	9 (2.0)		
	GI bleeding	44 (3.5)	2 (2.7)	18 (2.5)	24 (5.4)		
	Neurologic (NOS)	41 (3.3)	6 (8.0)	27 (3.8)	8 (1.8)		
	Else	38 (3.1)	1 (1.3)	23 (3.2)	14 (3.1)		
	Respiratory (NOS)	31 (2.5)	4 (5.3)	17 (2.4)	10 (2.2)		
	Renal/metabolic	24 (1.9)	2 (2.7)	20 (2.8)	2 (0.5)		
	GI (NOS)	23 (1.9)	3 (4.0)	6 (0.8)	14 (3.1)		
	Aspiration pneumonia/poisoning/toxicity	23 (1.9)	3 (4.0)	16 (2.2)	4 (0.9)		
	Seizure	21 (1.7)	2 (2.7)	18 (2.5)	1 (0.2)		
	Neoplasm	12 (1.0)	1 (1.3)	7 (1.0)	4 (0.9)		
	Cardiovascular (NOS)	11 (0.9)	0 (0)	3 (0.4)	8 (1.8)		
	Drug overdose	10 (0.8)	1 (1.3)	7 (1.0)	2 (0.5)		

^a^Unplanned ED revisit in 72 hours; ^b^readmission in 14 days; ^c^comparison by Wilcoxon’s rank sum test. APACHE, acute physiology and chronic health evaluation; ED, emergency department; ICH/SAH/SDH, intracerebral hemorrhage/subarachnoid hemorrhage/subdural hemorrhage; CAD, coronary artery disease; CHF, congestive heart failure; COPD, chronic obstructive pulmonary disease; GI, gastrointestinal; NOS, not otherwise specified.

Of the 1,242 patients, 75 left the ED with missing outcomes because they were either transferred out or discharged against medical advice. Nonetheless, this population was homogenous to the original study population in baseline characteristics. Moreover, 448 (36.1%) patients died in the first 21 days on mechanical ventilation. There were no statistically significant differences between survivors and nonsurvivors for age, triage level, unplanned ED re-visit in 72 hours, re-admission in 14 days, hours from ED arrival to ventilator connection, and length of ED stay. There were significant differences between the survivors and nonsurvivors for sex, APACHE II scores, hours from ventilator connection to leaving the ED, and principal diagnostic categories.

The estimated HRs of ICU waiting time on mortality in each subset (by ICU waiting hours) revealed that in the subsets of ‘<2 hours’, ‘<3 hours’ and ‘<4 hours there was no significant difference in mortality among them for each additional hour of waiting (Table 2). Thus, it was inferred that with an ICU waiting time of <4 hours, every additional 1 hour waiting had no impact on mortality. In contrast, in the subsets of ‘<5 hours’, ‘<6 hours’, ‘<7 hours’ , ‘<8 hours’ , ‘<9 hours’ , and ‘<10 hours’ , each additional hour of waiting in each subset imposed a significant difference on the probability of death. The time effect on mortality emerged between hours 4 and 5 so hour 4 was proposed as the demarcation of delayed and not-delayed.

**Table 2:** **Effects of ICU waiting time on mortality in the first 21 ventilator days by subsets of different waiting hours**

**Population subset by different ICU-waiting hours**	**HR** ^***a***^ **(95% CI)**	***P*** **value**
Less than 2 hours (n = 118)	0.64 (0.28-1.48)	0.297
Less than 3 hours (n = 240)	1.25 (0.92-1.71)	0.147
Less than 4 hours (n = 337)	1.17 (0.98-1.39)	0.093
Less than 5 hours (n = 420)	1.15 (1.02-1.31)	0.026
Less than 6 hours (n = 473)	1.14 (1.03-1.26)	0.008
Less than 7 hours (n = 524)	1.13 (1.04-1.21)	0.002
Less than 8 hours (n = 561)	1.11 (1.04-1.18)	0.001
Less than 9 hours (n = 591)	1.07 (1.01-1.13)	0.017
Less than 10 hours (n = 622)	1.03 (1.00-1.05)^*b*^	0.043
Less than 11 hours (n = 657)	1.02 (0.98-1.06)	0.354
Less than 12 hours (n = 681)	1.01 (0.97-1.04)	0.772
Less than 24 hours (n = 879)	0.99 (0.98-1.01)	0.436
Entire waiting hours (n = 1242)	0.99 (0.99-1.00)^*b*^	<0.001

^a^HR of ICU waiting hour, model of multivariate Cox regression, adjusted for sex, ICU waiting hour, APACHE II score, triage, unplanned ED revisit in 72 hours and readmission in 14 days; ^b^not satisfied by the proportional assumption of Cox model, assessed by Kolmogorov-type supremum test. ICU, intensive care unit; HR, hazard ratio; CI, confidence interval; ED, emergency department; APACHE, acute physiology and chronic health evaluation.

Thus, a wait of up to 4 hours was considered as ‘non-delayed’, whereas a wait longer than 4 hours was ‘delayed’. Using this to reassess the effects of delayed admission on mortality and prolonged hospital stay, multivariate logistic regression showed that higher APACHE II score (odds ratio (OR) 1.05, 95% confidence interval (CI) 1.03 to 1.07), triage level as non-urgent (OR 1.66, 95% CI 1.12 to 2.46), and delayed ICU admission (OR 1.41, 95% CI 1.05 to 1.89) were associated with increased probability of death within the first 21 ventilator days (Table 3). Furthermore, to ensure that the different groups had similar initial severity scores and expected mortality, the non-delayed and delayed groups were matched based on the same APACHE II score in a 1:2 ratio. The post-matched multivariate logistic regression, with adjustments for the same variables as Table 3, showed that delayed ICU admission (OR 2.87, 95% CI 2.05 to 4.04) was still associated with increased mortality.

**Table 3:** **Multivariate logistic regression of mortality and prolonged hospital stay**

**Variables**	**Mortality** ^***a***^		**Prolonged LOS** ^***b***^	
	**OR** ^***c***^ **(95% CI)**	***P*** **value**	**OR** ^***a***^ **(95% CI)**	***P*** **value**
APACHE II score	1.05 (1.03-1.07)	<0.001	1.03 (1.01-1.06)	0.012
Male sex	1.17 (0.90-1.52)	0.236	1.04 (0.75-1.44)	0.818
Level^*d*^ >2 vs. level 1	1.66 (1.12-2.46)	0.019	1.04 (0.63-1.70)	0.846
Level^*d*^ 2 vs. level 1	1.37 (0.93-2.02)	0.682	1.15 (0.70-1.90)	0.504
Delayed vs. non-delayed^*e*^	1.41 (1.05-1.89)	0.024	1.56 (1.07-2.27)	0.020

^a^Mortality in the first 21 ventilator days; ^b^length of stay >30 days; ^c^adjusted principal diagnostic categories; ^d^triage level; ^e^admission to intensive care unit. LOS, length of stay; OR, odds ratio; CI, confidence interval; APACHE, acute physiology and chronic health evaluation.

**Figure 2:**
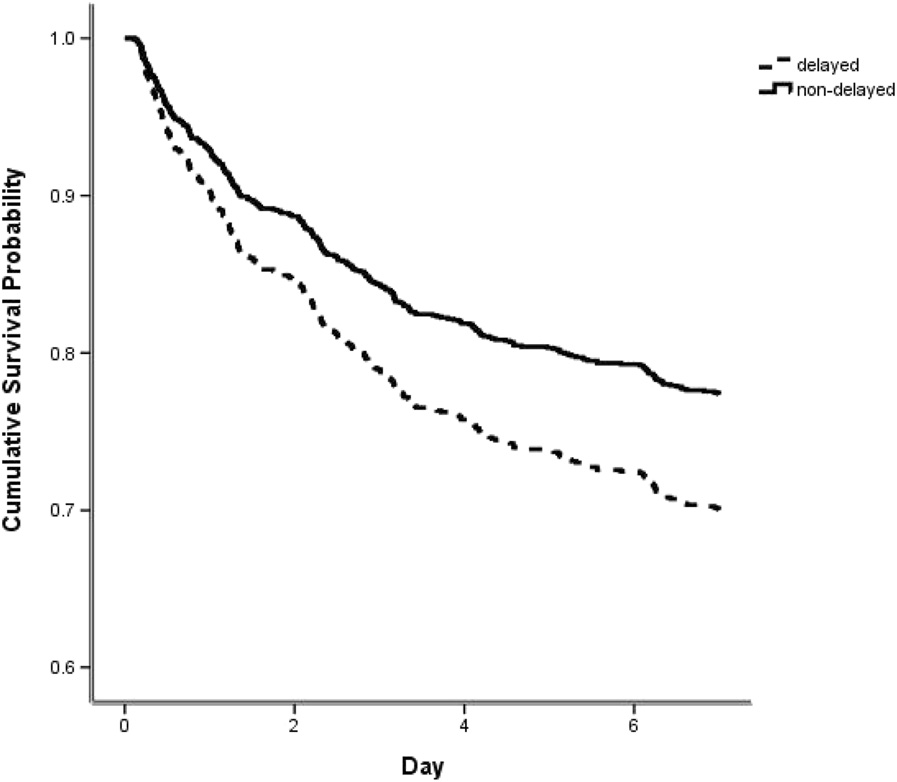
**Curves of cumulative survival in seven ventilator days.**

Comparing cumulative survival in the first seven ventilator days to evaluate the effect on the short-term mortality, delayed admission had an impact on mortality within the first seven ventilator days (Figure 2).

Of the 719 patients who survived the first 21 ventilator days, 327 had prolonged hospital stay. Multivariate logistic regression revealed that APACHE II score and delayed ICU admission had an odds ratio (OR) of 1.03 (95% CI, 1.01 to 1.06) and 1.56 (95% CI, 1.07 to 2.27), respectively (Table 3). Sex and triage showed no association with prolonged hospital stay.

Mortality across the principal diagnostic categories was compared using multivariate Cox regression and the analysis was adjusted for factors of sex, entire ICU waiting time at the ED, APACHE II score, and triage level (Table 4). Using the most prevalent diagnostic category (respiratory infection) as a reference, the category of seizure was associated with better survival in the first 21 ventilator days (HR 0.11, 95% CI 0.02 to 0.81). The categories of sepsis (HR 1.74, 95% CI 1.40 to 2.17), post-cardiac arrest (HR 2.06, 95% CI 1.43 to 2.96), and gastrointestinal bleeding (HR 2.88, 95% CI 1.69 to 4.92) were associated with poorer survival in the first 21 ventilator days.

**Table 4:** **Cox regression analysis of mortality in the first 21 ventilator days across the different diagnostic categories in multivariate analysis**

**Diagnostic categories**	**HR** ^***a***^ **(95% CI)**	***P*** **value**
Seizure disorder	0.11 (0.02-0.81)	0.030
Renal/Metabolic	0.37 (0.12-1.16)	0.089
CHF	0.65 (0.36-1.18)	0.154
COPD	0.65 (0.36-1.18)	0.158
Neurologic (NOS)	0.88 (0.47-1.62)	0.671
Aspirations pneumonia/poisoning/toxicity	0.90 (0.39-2.03)	0.790
CAD	0.94 (0.59-1.50)	0.781
Respiratory infection	1 (reference)	
ICH/SAH/SDH	1.31 (0.94-1.84)	0.112
Drug overdose	1.41 (0.35-5.72)	0.632
Respiratory (NOS)	1.45 (0.80-2.61)	0.219
Else	1.55 (0.91-2.64)	0.108
Neoplasm	1.57 (0.76-3.21)	0.222
Sepsis	1.74 (1.40-2.17)	<0.001
Post-cardiac arrest	2.06 (1.43-2.96)	<0.001
Gastrointestinal bleeding	2.88 (1.69-4.92)	<0.001

^a^Derived from multivariate Cox regression; had adjusted sex, ICU waiting time, APACHE II score, triage level. HR, hazard ratio; CHF, congestive heart failure; COPD, chronic obstructive pulmonary disease; NOS, not otherwise specified; CAD, coronary artery disease; ICH/SAH/SDH, intracerebral hemorrhage/subarachnoid hemorrhage/subdural hemorrhage.

## Discussion

Critically ill patients need both intensive and longitudinal care from their physicians and nursing staff. Unfortunately, the EDs are often crowded with patients waiting for triage, initial treatment, and stabilization. The stay of critically ill patients intensifies ED overcrowding and these conditions hinder the ED from providing continuous optimal care. Moreover, EDs are limited by their initial design and purpose, and are not as well resourced as ICUs. The ED can accommodate critically ill patients temporarily, but the care provided cannot replace that of the ICU. Many studies have investigated this situation [3,16–19].

In the ED of the study hospital, even though the ventilated patients were monitored by various portable equipment and taken care of by ED staff members who were also certified in providing critical care, there were many limitations in performing critical care in the ED. The ratios of nurse-patient or doctor-patient usually exceeded those of the ICU. Patients’ relatives and attendants were invited to collaborate in some of the nursing care work like feeding via nasogastric tube, and input/output measurements and recording. The narrow ED stretchers also made it difficult to care for decubitus ulcer prevention, and oral and perineum hygiene. These made the control of nosocomial infection difficult and complex.

For the most part, the stay of critically ill patients in the ED is attributed to insufficient ICU capacity [3]. Although the disposition to admit to the ICU is made after initial treatment and stabilization, ventilated patients have to wait and be cared for at the ED. In the study hospital, the ventilated patients in the ED are transferred to the ICU based on the principle of ‘first come, first served’. Under this operational system, there is no significant correlation between the ICU waiting time and the severity of illness in the patients (Table 5). Thus, the bias of patient selection due to ICU preference is not significant.

**Table 5:** **Correlation matrix of the time of different phases of ED stay and APACHE II scores (n = 1,242)**

	**APACHE II score**	**Door to ventilator**	**Ventilator to leaving ED**	**LOS in ED**
APACHE II score		-0.18**	0.05	-0.09*
Door to ventilator^*a*^			-0.03	0.68**
Ventilator to leaving ED^*b*^				0.68**
LOS in ED^*c*^				

^a^Door to ventilator, time between arrival at the ER and connecting to the ventilator; ^b^ventilator to leaving ED, time between connecting to the ventilator and leaving the emergency department; ^c^LOS in ED, length of stay in the ED. ^*^ *P* <0.005, ^**^ *P* <0.0005. ED, emergency department; APACHE, acute physiology and chronic health evaluation; LOS, length of stay.

Prior researches have studied the effects of ICU waiting time on patient outcome [3,8,10,11,20,21]. However, most of these investigations treated ICU waiting time in a dichotomous manner and the definition of ‘delayed admission’ varied across studies. Few studies measured the length of ICU waiting time as a continuous variable [12,21], and none answered the question ‘How long a wait is considered delayed?’. Furthermore, most studies do not count the adverse events before ICU admission while waiting at the ED and simply enrolled patients who are admitted to the ICU, that is, those who survive their ED wait for ICU admission. Furthermore, multi-hospital studies are limited by heterogeneity of treatment ability and quality between hospitals, heterogeneity of study populations, and by lack of data on patients’ disease severity or APACHE II scores.

**Figure 3:**
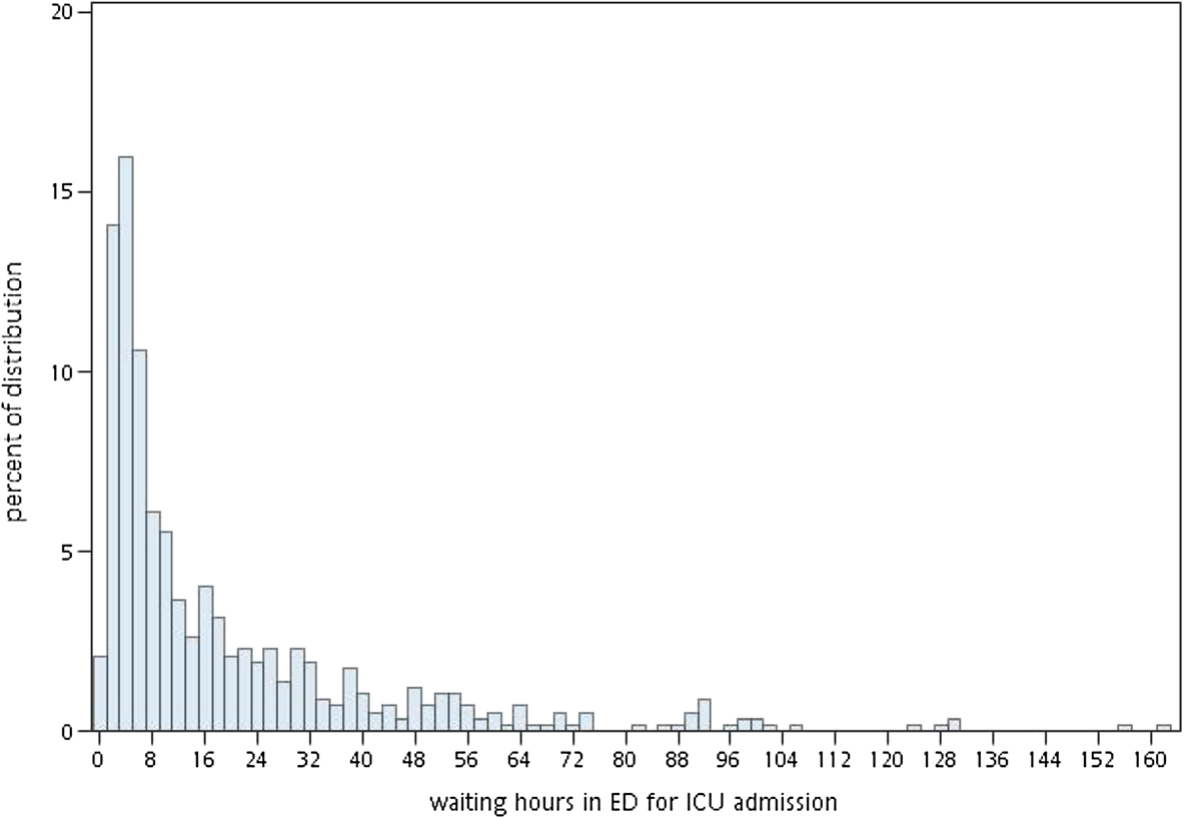
**Distribution of deaths by ICU waiting hours at the ED.** Percentage = (number of deaths in 21 ventilator days in the group with the designated ICU waiting hours)/(number of all deaths in 21 ventilator days).

The present study defined the cutoff time based on the emergence of statistical differences in the waiting time variable. The HR of ICU waiting time did not become statistically significant until after 4 hours. Thus, an ICU waiting time of <4 hours was non-delayed. But admitting such patients to the ICU in the non-delayed phase did not hint of whether they were safe or would survive. The fact is just that for each additional waiting hour in this phase, there was no significant difference added to mortality. The proposed cutoff point by this study is earlier than that used in current practice [10], perhaps because the study considered deaths that occurred during the wait at the ED before ICU admission. Although the time effect was no more statistically significant after 10 hours, this might be explained by the distribution of deaths by ICU waiting hours at the ED (Figure 3). The distribution of deaths by ICU waiting time is right-skewed, meaning that most deaths have occurred in the first ICU waiting hours. As such, the time effect in the regression model will return to neutral in view of the whole length of ED wait for ICU admission.

Non-urgent triage results are associated with increased hazards (Table 3), which may be related to the latent clinical course of some diseases and results in a prolonged waiting time for the physician’s visit, especially in an overcrowded ED. The results here for triage are similar to those of a previous study of trauma patients wherein triage results are associated with deleterious effects from the ED to the ICU [22].

### Study limitations

First, in the study hospital, ventilator hours in the ED were taken as the length of ICU waiting. This was because the ICU booking was made after the patient was connected to a ventilator. Caution should be taken when extending this model to other hospitals where the ICU booking is much earlier than ventilator connection, or where alternative respiratory support devices are used to replace the ventilator. Under such conditions, the hypothesis would be that every additional hour in the first 4 ventilator hours does not have an impact on mortality. Nonetheless, further research is suggested.

Second, the present research is restricted by its retrospective study design. Some data have not been accessed due to information attrition. Conditions that are not recorded in the charts cannot be adjusted in the statistical model, including the severity of ED overcrowding (for example, patient count, occupancy the ED and ICU) [23,24] or resources of the nursing staff [25,26].

Third, although delayed ICU admission is associated with higher mortality and more resource utilization, the causes of delayed ICU admission have not been explored and what was deferred in the ED while waiting for the ICU admission was not known. These may also affect outcomes. Further studies are warranted to investigate the etiology of delayed ICU admission and what treatments or processes are deferred or slowed down during the ICU waiting time at the ED.

Fourth, the present research is a single hospital study. Prospective studies with a multiple-center focus and adjustments for potential confounding variables are warranted.

## Conclusions

The ED is unable to offer a sustained optimal care to critically ill patients. Such patients may suffer adverse outcomes if they are not promptly admitted to the ICU. Delays in such admission may increase the probability of mortality and also results in additional resource utilization if they survive. For ventilator patients in the ED, a benchmark waiting time of no more than 4 hours for ICU admission is recommended. Further prospective, multicenter investigations are recommended.

## Key messages

- The proposed definition of non-delayed ICU admission is that ED waiting (by hours) does not statistically increase mortality.
- Delayed ICU admission of mechanically ventilated patients in the ED is associated with higher mortality and additional resource utilization.
- The ED waiting time for ICU admission should have a cutoff time of no more than 4 hours as a benchmark.

## Abbreviations

APACHE: acute physiology and chronic health evaluation
CAD: coronary artery disease
CHF: congestive heart failure
COPD: chronic obstructive pulmonary disease
ED: emergency department
GI: gastrointestinal
ICH/SAH/SDH: intracerebral hemorrhage/subarachnoid hemorrhage/subdural hemorrhage
ICU: intensive care unit
IHCA: in-hospital cardiac arrest
LOS: length of stay
NHI: National Health Insurance
NOS: not otherwise specified
OHCA: out-of-hospital cardiac arrest
ROSC: return of spontaneous circulation
